# Skeletal Phenotyping of Period‐1‐Deficient Melatonin‐Proficient Mice

**DOI:** 10.1111/jpi.70020

**Published:** 2024-12-19

**Authors:** Olaf Bahlmann, Shahed Taheri, Manuela Spaeth, Katrin Schröder, Arndt F. Schilling, Christian Dullin, Erik Maronde

**Affiliations:** ^1^ Faculty of Medicine Institute for Anatomy II Goethe University Frankfurt Frankfurt am Main Germany; ^2^ Department of Trauma Surgery, Orthopaedic Surgery and Plastic Surgery University Medical Center Göttingen Göttingen Germany; ^3^ Faculty of Medicine Institute for Cardiovascular Physiology Goethe University Frankfurt Am Main Germany; ^4^ Department for Diagnostic and Interventional Radiology University Medical Center Göttingen Göttingen Germany; ^5^ Translational Molecular Imaging, MPI for Multidisciplinary Sciences Göttingen Germany; ^6^ Department for Diagnostic and Interventional Radiology University Hospital Heidelberg Heidelberg Germany; ^7^ Elettra‐Sincrotrone Trieste SCpA, Basovizza Trieste Italy

**Keywords:** bone density, clock genes, microcomputed tomography, period‐1; body size regulation; ossification

## Abstract

In mice, variability in adult bone size and density has been observed among common inbred strains. Also, in the group of genes regulating circadian rhythmicity in mice, so called clock genes, changes in body size and skeletal parameters have been noted in knockout mice. Here, we studied the size and density of prominent bones of the axial and appendicular skeleton of clock gene *Period*‐1‐deficient (*Per*1^‐/‐^) mice by means of microcomputed tomography. Our data show shorter spinal length, smaller and less dense femora and tibiae, but no significant changes in the shape of the skull and the length of the head. Together with the significantly lower total body weight of *Per*1^‐/‐^ mice, we conclude that *Per*1‐deficiency in a melatonin‐proficient mouse strain is associated with an altered body phenotype with smaller appendicular (hind limb) bone size, shorter spine length and lower total body weight while normal head length and brain weight. The observed changes suggest an involvement of secondary bone mineralisation with impact on long bones, but lesser impact on those of the skull. Evidence and overall physiological implications of these findings are discussed.

## 1 Introduction

In mice, there is large genetic variability in the skeletal phenotype among common inbred strains [9, 12]. There are also significant changes of bone structure and density with ageing [58]. The genetic architecture of this skeletal phenotype, which also influences body size in mammals is complex and includes extended portions of the transcriptome [37].

Also genes regulating circadian rhythmicity, the so‐called clock genes [1, 40], influence adult body weight as well as bone and skeletal phenotypes [10, 19, 23, 32, 39, 45, 52, 56, 61, 62, 68, 69, 70, 71]. Some of these changes may be due to the expression of a circadian clock in cartilage [29]. There seems to be an interplay between clock genes and rhythmic melatonin production [25]. This is important, when we try to transfer findings in mice to humans, as in contrast to humans, some mouse strain do not produce melatonin [41, 51].

The specific roles of clock gene inactivation, including *Per*1, have been studied extensively before. This includes cell specific expression in the skeletal system during development and the resulting physiological interactions [39, 69]. However, the specific role of *Per*1 in a melatonin proficient mouse model has not been investigated.

Mammalian *Period*‐1 (*Per*1) was discovered together with its homologues *Per*2 and *Per*3 in the late 1990s [2, 63, 80]. When *Per*1‐deficient mice became available, its influence on the circadian and diurnal clock properties was studied [4, 15, 75]. After the first analyses of the promotor region [31, 48], data on the transcriptional regulation of *Per*1 [8, 44, 47, 48] became available. This established *Per*1 as a kind of immediate early gene modulated by many different signalling pathways [7, 8, 47, 48] and *Per*1 has since been shown to be involved in many biological processes [39]. However, most of these early reported phenotypes were from mouse strains that do not produce melatonin rhythmically [27, 36, 41].

In a melatonin‐deficient background, *Per*1‐deficient mice (m*Per*1^Brdm1^) [75] did not show a bone density phenotype [23].

Nevertheless, in both melatonin‐deficient and ‐proficient *Per*1‐deficient mice, a wide variety of physiological parameters were described as altered. These include changed tibialis anterior muscle strength [5], altered glucocorticoid level and body growth characteristics [19] or lower reproductive success with signs of premature ageing [10, 39, 52, 53]. Also, the non‐redundancy of *Per*1 with its paralog *Per*2 has been shown from the adult in vivo phenotype down to the cellular level [4, 5, 45, 50, 75].

Here, we describe the bone and skeletal phenotype of mice with a deletion of the *Per*1 gene in the melatonin‐proficient C3H‐background [6, 10, 17, 55, 64] and compare them with C3H‐wildtype and outline possible explanations for the observed phenotypes.

Size and density of prominent bones of the axial (head), and appendicular (hind limbs) skeleton as well as spine length were studied by means of micro‐CT in *Per*1^‐/‐^ mice backcrossed into the melatonin‐proficient C3H background.

Moreover, the weights of inner organs and the brain in *Per*1^‐/‐^ mice were compared to the wildtype.

Evidence, overall physiological implications and limitations of the study and these findings are discussed.

## 2 Materials and Methods

### 2.1 Animals

All experiments reported here were conducted in accordance to the guidelines of the European Communities Council Directive (89/609/EEC) for humane animal care.

*Per*1‐deficient (129S‐*Per*1^tm1Drw/J^ = *Per*1^‐/‐^) mice [4] were bred onto a melatonin‐proficient C3H genetic background for at least 14 generations (Jackson nomenclature of the backcrossed mice is C3.129S‐*Per*1^tm1Drw/J^) as described before [6, 10, 17, 41, 55, 64] and compared with age‐matched C3H wildtype mice (WT) purchased from JanvierLabs (France) born and raised in the same animal facility. Genotype monitoring was done by PCR. Health status of the animals was monitored regularly as described and did not differ between the genotypes. Details on the general habitus of these mice and their behaviour have been published before [10, 55].

### 2.2 Methods

Mice were sacrificed in or after the 12th week after birth (WT, aged 82 and 99 days, 3 and 4 mice, respectively) and (*Per*1^‐/‐^, aged 84 and 94 days, 2 and 4 mice, respectively). Perfusion fixation was carried out as described previously [35]. After immersion fixation in 4% paraformaldehyde, the storage solution was changed to 70% alcohol before micro‐CT analysis.

Microcomputed tomography (micro‐CT = µCT), of whole specimens as well as the computer‐assisted analyses thereof were done as described [6].

### 2.3 Bone Parameter Determination

Bone length, volume and density and comparison of the skull shape (see also below) were done using VGStudio Max 3.1 in combination with MeshLab [https://www.meshlab.net] as described by [49]. Spine length was determined for those 36 segments beginning with the atlas that were measurable in all specimen analysed.

### 2.4 Skull Form Comparison

To compare the shape of two skulls both data sets have been manually co‐registered in space. The surface of the skulls is represented as a mesh containing points and associated triangles. To calculate the local deviation between the skulls we used the Hausdorff distance, meaning for each point on the surface mesh of one skull the distance to the closest point of the mesh of second skull is calculated. Therefore, Hausdorff distance is always positive. Similar procedures have been used before to detect variation of skull size in shrews, moles and minks [20, 49, 54].

### 2.5 Organ Weight Comparison

To quantify the sizes of different organs in the *Per*1^‐/‐^ mice in comparison to WT, cardially perfused and PFA‐fixed specimens (brain, heart, liver, and kidneys) were dissected and the wet weight determined (Sartorius analytical balances, BP221S).

### 2.6 Statistics

Data were tested for normal distribution by Shapiro‐Wilk or Kolmogorov‐Smirnov tests. Genotypes were analysed using Student's t‐test as implemented in GraphPad Prism (GraphPad, San Diego, CA, USA, Vs. 7.02/8.4). The criterion of significance was *p* ≤ 0.05.

### 2.7 Data Access

We created a public archive for the raw data (DOI 10.17605/OSF.IO/REB56; https://osf.io/reb56/).

## 3 Results

**FIGURE 1:**
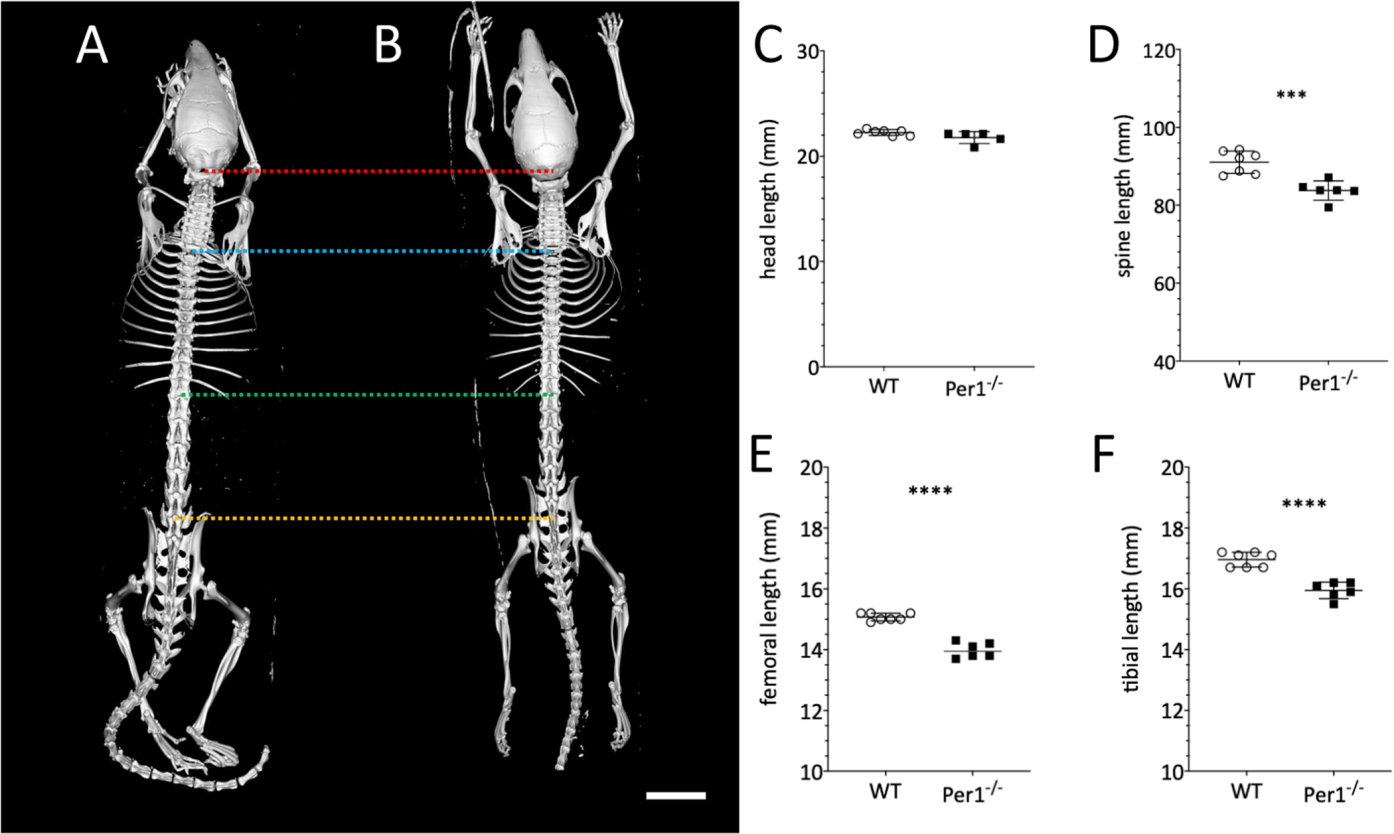
Images of whole body microtomographical scans (μCT; dorsal view) of a WT (A) and a *Per*1^‐/‐^ animal (B). The images are derived from videos with a 360° panoramic view, which can be found in the supplementary materials. The scale bar is 10 mm. For orientation in the µCT images, the upmost (red) line marks the beginning of the cervical part of the spine (atlas) in WT, the blue line the end of the cervical and beginning of the thoracal part, the green line the end of the thoracal and beginning of the lumbal part and the orange line the end of the lumbal part. The right part of the image shows the comparison of head (C), spinal (vertebral column; (D), femoral (E) and tibial (F) length. The statistical analysis was done by *t*‐test (*N* = 6 WT; *N* = 7 *Per*1^‐/‐^; *p* < 0.001***; *p* < 0.0001****).

The basis of our investigation of the whole body/skeletal phenotype is microcomputed tomography (micro‐CT, µCT) data from WT and *Per*1^‐/‐^ mice. Figure 1 displays the dorsal view of the skeleton of one representative specimen of each a WT (A) and a *Per*1^‐/‐^ (B) male animal. Whereas head length was not significantly different between WT and *Per*1^‐/‐^ mice (Figure 1C), the spinal column (Figure 1D; *p* < 0.001***) as well as femur (Figure 1E; *p* < 0.0001****) and tibia (Figure 1F; *p* < 0.001****) were significantly shorter in *Per*1^‐/‐^ mice.

**FIGURE 2:**
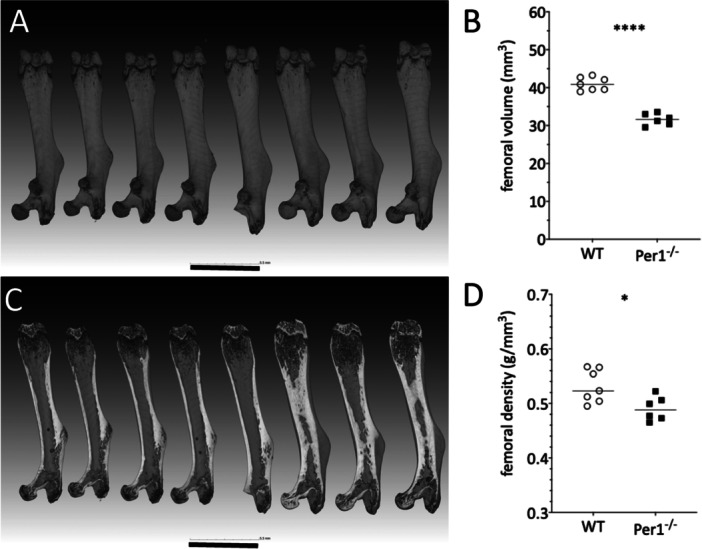
Figure 2 shows images of the femoral μCT of four *Per*1^‐/‐^ (left) and four WT C3H (right side) mice. The upper images (A) display the surface view of the femora, the lower picture (C) a mid‐section view illustrating the relative cortical and spongiosal areas. The scale bar represents 5.5 mm. Statistical analysis of femoral volume (B) and femoral bone density (D) was done by *t*‐test (*N* = 6 WT; *N* = 7 *Per*1^‐/‐^; *p* < 0.05*; *p* < 0.0001****).

Microtomographical analysis further showed that the femora (Figure 2A/C) of WT mice were significantly longer (Figure 1E; *p* < 0.0001****) and had higher bone volume (Figure 2B; *p* < 0.0001****) as well as a higher bone density (Figure 2D; *p* < 0.05*) compared to *Per*1^‐/‐^ mice.

**FIGURE 3:**
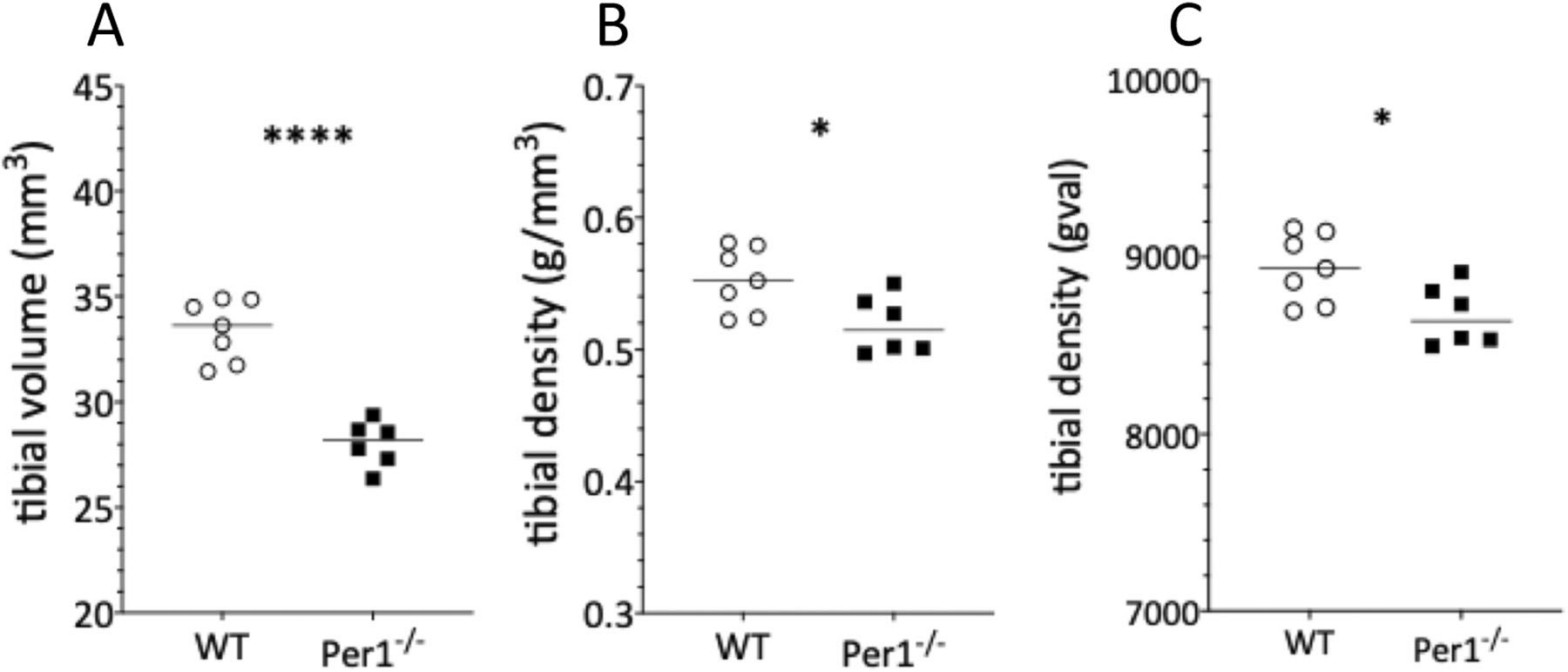
Figure 3 shows tibial volume (A), and density as measured in g/mm^3^ (B) or gval (C) which differ significantly in *Per*1^‐/‐^ in comparison to WT (*p* < 0.05*, *t*‐test). The mean difference in volume is 16.2% + 3.9% (SD). Statistical analysis was done by *t*‐test (*N* = 6 WT; *N* = 7 *Per*1^‐/‐^; *p* < 0.05*; *p* < 0.0001****).

*Per*1^‐/‐^ mice showed a mean reduction in bone volume of 23% ± 4.9% (SD) in femur and 16.2% ± 3.9% (SD) in tibia (Figure 3A), whereas length was reduced by 7.2% + 1.8% (SD) in femur (Figures 1E) and 5.9% + 1.7% (SD) in tibia (Figure 1F). Bone density, both measured as gval or g/mm^3^, differed significantly in both femur (Figure 2D; *p* < 0.05*) and tibia (Figure 3B/C; *p* < 0.05*) between the two genotypes.

**FIGURE 4:**
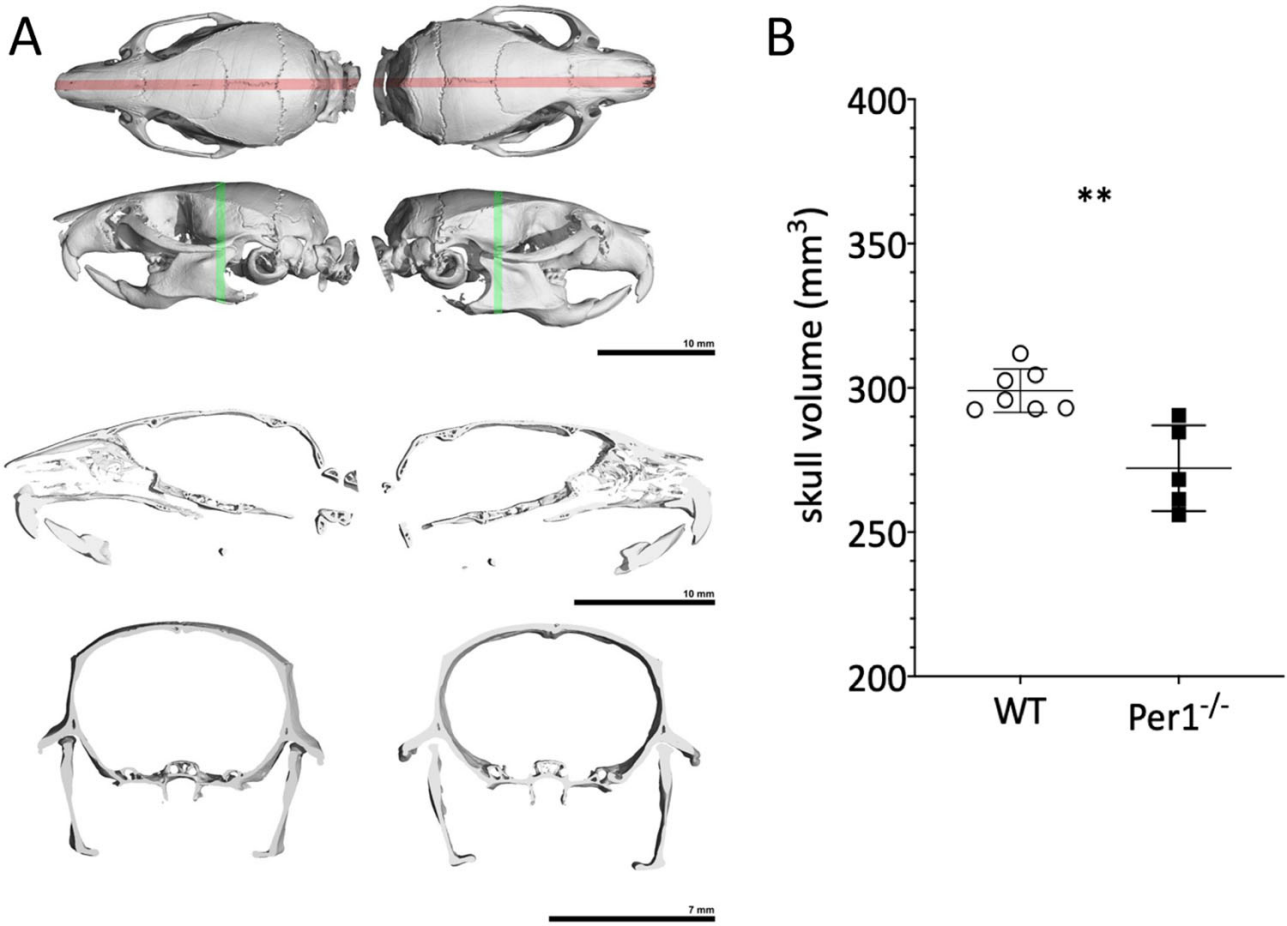
Figure 4 shows an example of the overall µCT parameters as visualised in top (red line) and side (green line) views of the whole skull as well as a sagittal (third row) and frontal (lower row) section (A). Left side of A shows WT and the right side *Per*1^‐/‐^. Side bars indicate 10 mm for the total and sagittal views and 7 mm for the frontal view. (B) shows the statistical comparison of skull volume between WT and *Per*1^‐/‐^ (WT *N* = 7; *Per*1^‐/‐^ *N* = 6, *p* < 0.01** *t*‐test).

Whole head/skull analysis revealed that although WT and *Per*1^‐/‐^ looked generally very similar (Figure 4A) and head length did not differ (Figure 1C), skull volume was significantly smaller in *Per*1^‐/‐^ animals (Figure 4B; *p* < 0.01**).

**FIGURE 5:**
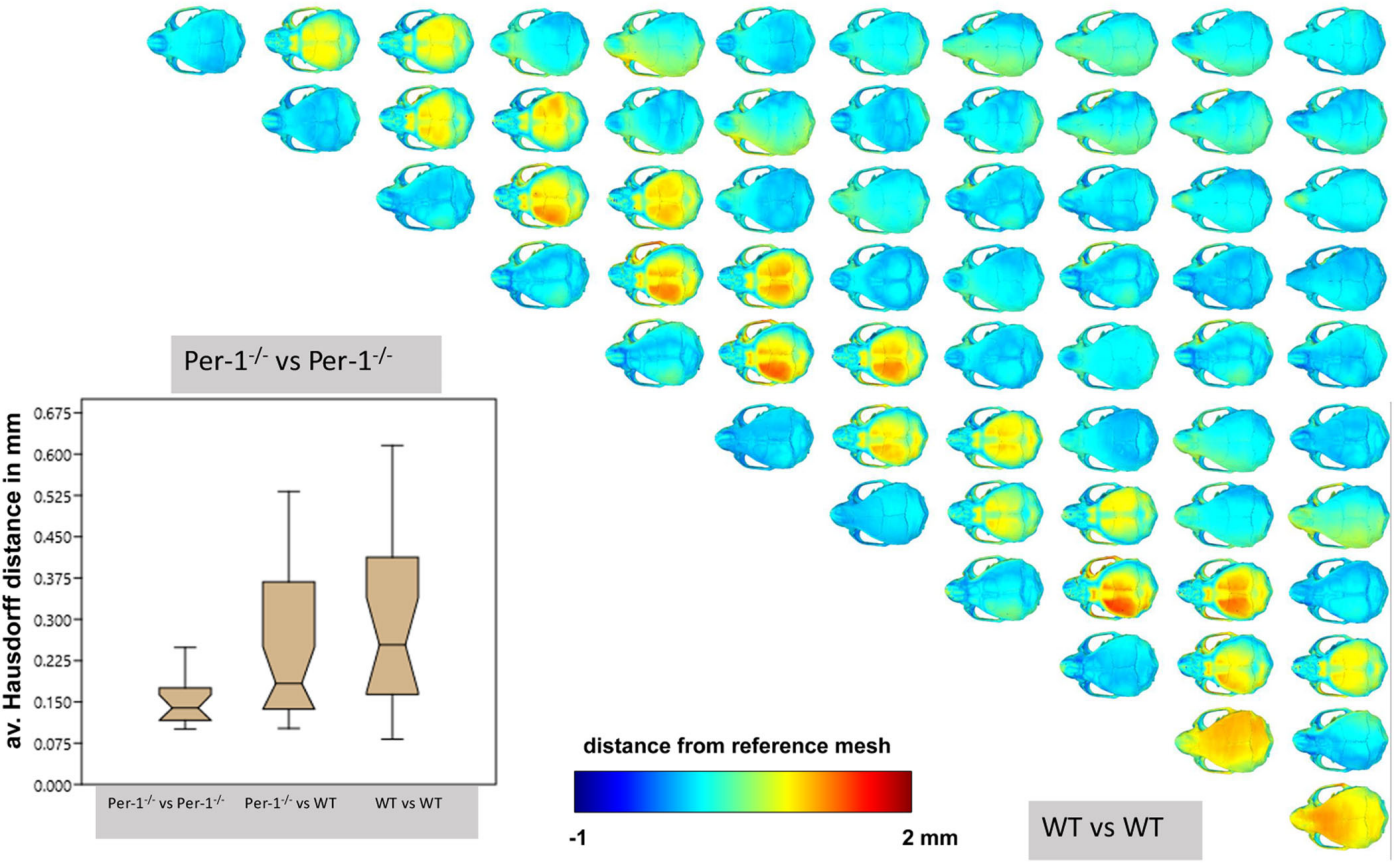
Figure 5 compares all skulls of WT and *Per*1^‐/‐^ animals by a 1:1 analysis of every animal. The deviation in skull shape was in a low range (−1 to +2 mm) and not significantly different between WT and *Per*1^‐/‐^ animals regarding Hausdorff distance.

Next, we compared the skull shape of WT and *Per*1^‐/‐^animals (Figure 5) by a 1:1 analysis of every animal. The deviation in skull shape was in a low percent range (−1 to +2 mm) and was neither significant inside the wildtype (WT vs. WT) and the *Per*1^‐/‐^ animals (*P* vs. *P*) nor between the genotypes (WT vs. *Per*1^‐/‐^) as also shown by the Hausdorff distance analysis in the inserted graph.

No difference in the head length but in skull volume and appendicular skeletal bones raised the question of brain and other organ size/weight measurements.

**FIGURE 6:**
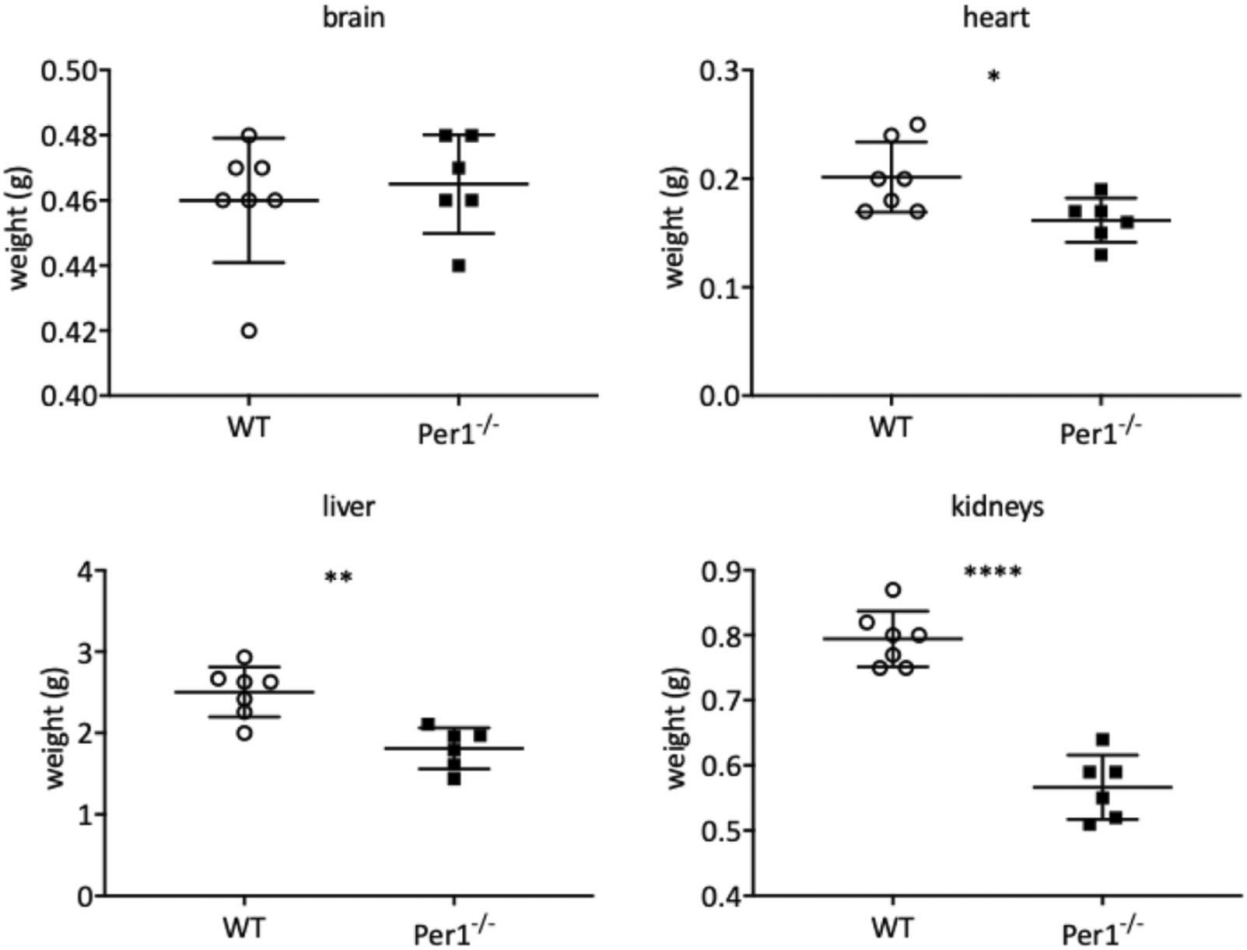
Figure 6 shows the organ weights in WT and *Per*1^‐/‐^ male mice (WT, *N* = 7) and (*Per*1^‐/‐^, *N* = 6). Note that the brain weight did not significantly differ between *Per*1^‐/‐^ and WT animals. However, the weights of heart (*p* < 0.05*), liver (*p* < 0.01**) and kidneys (*p* < 0.0001****) were significantly different between *Per*1^‐/‐^ and WT animals.

Brain weight did not significantly differ between *Per*1^‐/‐^ and WT animals (Figure 6; upper left side). However, the weights of heart (*; *p* < 0.05; Figure 6 upper right side), liver (**; *p* < 0.01; Figure 6 lower left side) and kidneys (****; *p* < 0.0001; Figure 6 lower right side) were significantly lower in the *Per*1^‐/‐^ animals.

## 4 Discussion

The *Period*‐1 gene and its protein product PER‐1 have been shown to influence a multitude of parameters from the cellular, the tissue/organ and organismic level up to behaviour [16, 39, 50, 69]. Previous observations on the strength of the tibialis muscle [5], structure of the larynx [6], endocrine bone‐related signalling [19, 32], reproduction [52, 53] and natural behaviours [10] suggest that the observed differences may result from a generalised underlying difference in whole body structure [16, 39]. Indeed, differential gene expression analysis undertaken in brain tissue of *Per*1^‐/‐^ mice in comparison to WT revealed changes in expression of many growth‐related genes [6, 55].

The first investigation of bone in *Per*1^‐/‐^ mice did not detect a significant bone density phenotype [23, 24]. This was surprising, since *Per*1 was subsequently shown to be transcriptionally activated in pre‐osteoblastic and pre‐chondroblastic cells by parathyroid hormone treatment (PTH) [30, 32]. Furthermore, *Per*1 oscillates in a circadian manner in mesenchymal stem cell, bone and tooth tissue [28, 29, 33, 34, 42, 76, 77, 79]. In addition to these findings, specific roles for *Per*1 have been described in growth plate development, enchondral ossification [61] and bone resorption [62]. Circadian rhythms in cultivated calvarial bone were detectable over months using a *Per*1‐luciferase reporter gene construct [46]. Moreover, a role for glucocorticoids in the circadian expression of *Per*1 was described [26], an effect resembling the partial reestablishment of circadian rhythmicity by rhythmic dexamethasone treatment in bone marrow derived cells [60]. Other rhythmic factors also affect cartilage and bone tissue, among them circadian disruption [57], metabolism [43] and melatonin [25, 67, 73, 78].

The factor melatonin demands specific consideration because some experimental models like C57BL/6 mice are melatonin deficient, but corticosteroid responsive [38]. To analyse, if melatonin might be the missing link between *Per*1 expression and bone, here we used C3H backcrossed *Per*1^‐/‐^ mice that are melatonin proficient [6, 10, 17, 41, 55, 64]. Interestingly, another instance where a clock gene phenotype was changed after backcrossing into melatonin‐proficiency has previously been described for the Clock‐delta 19 mutation [59].

However, melatonin proficiency and deficiency are not the only differences between the S129 and C3H strains. Therefore, it is conceivable that also these other differences may play a part in the observed phenotype.

However, as melatonin and *Per*1 are both important elements of the circadian clock, and a clear difference between S129 and C3H in melatonin has been described [36] it is suggestive to assume a connection to the phenotype described here.

In the present study, we determined size and weight parameters to understand the whole‐body appearance of *Per*1^‐/‐^ mice in comparison to age‐ and sex‐matched WT animals. *Per*1^‐/‐^ mice have shorter hind limb bones (femur and tibia) as well as reduced bone volume and density in both. *Per*1^‐/‐^ mice also display shorter spine length in comparison to WT. However, data also show that the head lengths and brain weights of *Per*1^‐/‐^ mice do not differ, whereas the weights of inner organs like heart, liver and kidneys are significantly reduced, albeit to different organ‐specific extent. WT and *Per*1^‐/‐^ mice used here were sacrified in or after the 12th week after birth. Considering age differences in µCT, in male C57BL/6 mice, the rapid growth phase of femur length lasts for 12 weeks and is followed by maintenance of the peak thereafter [3]. In C57BL mice aging mice, cortical bone mineral density is similar between 12 and 18 weeks, while trabecular bone mineral density is lower [58]. However, though these data refer to C57BL/6 mice, they may give a hint on skeletal growth dynamics in other mouse strains.

In general, smaller body size parallels smaller organ size, however, reduced inner organ size without for example, smaller head size can be a sign of dwarfism [13, 21]. The main form of dwarfism in humans is achondroplasia which is mostly due to mutations in the fibroblast growth factor receptor type 3 (FGF3R). There may however also be organ specific reasons like altered occurrence and number of organ specific monocytes in *Per*1^‐/‐^ as has been described for the liver [65]. It should be noted that in addition to the vertebrae also the intervertebral disc height may contribute to the total length of the spine [22]. However, a potential contribution of the intervertebral discs to total spine length has not been investigated here.

The combined findings with similar skull shape and head length as well as smaller femora and tibiae plus shorter spine length suggest a disproportionate whole‐body phenotype in *Per*1^‐/‐^ mice compared to WT. The smaller skull volume together with the unchanged brain weight is somehow confusing. One possible explanation may be a partial independence of skull volume and brain size, or in other words the skull volume of *Per*1^‐/‐^ mice is still large enough to house a brain of WT size. The phenotype described here does not fit into the main categories described in a study on the skeletal phenotype of several 100 knockout mouse models based on the Lexicon Pharmaceuticals database [14]. However, as the authors state, some of the genes displaying bone and skeletal phenotypes were not included in the latter publication due to patent and commercial interests of the sponsoring company, Lexicon Pharmaceuticals [14].

When speculating about the mechanisms by which deleting *Per*1 can cause the observed changes, there are some possible pathways. Generally, there are two modes of bone formation, endochondral (indirect ossification via a cartilage precursor tissue mostly affecting long bones) and desmal (direct ossification out of mesenchymal cells forming mostly flat bones including the skull). The phenotype observed here looks like a cartilage problem. *Per*1 has been shown to be expressed in cartilage and there is a connection to matrix metalloproteinase 13 (MMP13) that can degrade cartilage [66]. Regarding the bone phenotype observed in *Per*1^‐/‐^ mice, collagen type I or other proteins of the organic matter of bone, calcium metabolism, differentiation processes from mesenchymal stem cells to mineralised bone, endocrine influences of these processes by hormones, muscular mechanics, and tendon strength or neuronal or vascular input may be involved [13, 69]. It may also be the combined effect of some or all these factors influencing each other [13, 21].

As mentioned above several disproportionate growth and size phenotypes are associated with fibroblast growth factor (FGF) family signalling in chondrocytes, which comprises of several receptors and even more ligands [11, 18]. Some of the altered parameters described in *Per*1^‐/‐^ mice before, like the lower tibialis muscle strength and the role of *Per*1 in parathyroid hormone signalling in bone development, may be a consequence of this difference in habitus [5, 32, 39]. Whether these differences in bone density in light of shorter bone length and smaller bone volume are physiologically or functionally relevant in terms of frequency of fractures in mice is unknown. For the clock gene *Per*1, there is evidence that a nine‐base‐pair deletion or single nucleotide polymorphisms (SNPs) are related to bone and skeletal parameters in goat [72] and human [74]. However, whether these constitute physiologically, or clinically relevant vulnerabilities of the skeletal systems associated with *Per*1‐gene SNPs or base deletions therein, plus a potential role of other strain differences, as well as a role for *Per*1 in human bone and skeletal development with therapeutical implications, remains to be determined.

In summary, size and density of prominent bones of the axial (head) and appendicular (hind limbs) skeleton were investigated by means of micro‐CT and found to be different between WT and *Per*1^‐/‐^ mice with smaller and less dense femora and tibiae and a shorter spine in *Per*1^‐/‐^ animals. Moreover, the weights of heart, liver, kidneys were lower in *Per*1^‐/‐^ mice compared to WT. However, the weight of the brain did not significantly differ. Finally, data presented here and from previous work [6] show no significant difference in the length of the skull.

Together with the significantly lower total body weight in *Per*1^‐/‐^ mice [10, 55], which was not observed for this age group (3 months) in earlier work in melatonin‐deficient mice [19], we conclude that *Per*1‐deficiency leads to a whole‐body phenotype with smaller appendicular bone size, lower total body weight while normal skull shape and head length. To explain the observed parameters in terms of the described effects of the missing of *Per*1, many potential factors may contribute.

## Author Contributions

OB prepared the animals, contributed and analysed data and wrote the manuscript, MS and KS contributed µCT data, ST, AS and CD contributed data, analysed data, contributed graphs/images and commented on the manuscript, EM designed the study, assisted in the analysis of the data and wrote the manuscript.

## Ethics Statement

The animal experiments were allowed under the veterinary regulations of Germany (Allowance by the State of Hessen, RG Darmstadt: V54‐19c 20/15 – FU/1045).

## Conflicts of Interest

The authors declare no conflict of interest.

## Data Availability

The data that support the findings of this study are openly available in OSFHome at https://osf.io/reb56/. The data that support the findings of this study are available from the corresponding author upon request.
